# Domestic

**Published:** 1841-07-24

**Authors:** 


### DOMESTIC.

We copied from the last number of the Western Journal of Medicine and Surgery, a case of Axillary Aneurism, which had been operated upon by Professor Gross—with the post mortem examination, death having occurred thirty days after the ligature of the subclavian artery. The paper of Prof. Gross, of whi h this operation constitutes the basis, exhibits concisely the peculiarities of 26 cases of Axillary Aneurism, for which the subclavian has been tied by other surgeons. This summary is so valuable as a statistical record, that we extract the most important parts.

*Cases of Axillary Aneurism for which the Subclavian Artery was Tied by other Surgeons.*—*Case* 1.—The first case in which an attempt to tie the subclavian artery, by cutting above the clavicle, was accomplished, is that of Mr. Ramsden, of London.* John Townly, a tailor, aged thirty-two, of very intemperate habits, and peculiarly unhealthy aspect, was admitted into St. Bartholomew's Hospital, Tuesday, November 2, 1809, on account of aneurism in the right axilla. The most prominent part of the tumor was about half as big as a large orange, and there was also much distension underneath the pectoral muscle, so that it was impossible to bring the elbow near the side of the body. The limb, although it retained its temperature, soon became œdematous, and the pulsation of the radial artery, already very faint, at length entirely ceased. On the evening of the twelfth day a dark spot appeared on the centre of the tumor, surrounded by inflammation, which threatened more extensive destruction of the skin. A farther postponement of the operation being deemed inadmissible, Mr. Ramsden performed it the next day, tying the artery on the outside of the scalene muscle. The shoulder being considerably elevated by the tumor, great difficulty was experienced in passing the ligature; this, however, was finally overcome by a probe of ductile metal and a pair of small forceps. As soon as the artery was tied, the pulsation in the swelling ceased; the limb afterwards fully retained its temperature; and the tumor had already somewhat diminished in volume, when about five days after the operation the patient expired. On examination, it was ascertained that the subclavian artery, where the ligature was applied, was so nearly separated that it was held together only by a few shreds ef dead matter. Each extremity of the vessel appeared to be completely consolidated and impervious, leaving no doubt of its being fully competent to resist the impetus of the blood from the heart. The aneurismal sac contained about two pints of blood, the greater part of it in a fluid state; the heart and large vessels were perfectly sound, even the subclavian, excepting at the side of the ligature; and the collateral vessels did not seem to have acquired any increase of capacity beyond what is natural to them.

*Case* 2.—In the year 1811, the subclavian artery was tied in the London Hospital, in a case of axillary aneurism, by Sir William Blizard. The patient, who was old and debilitated, died on the fourth day, though strong hopes were at first entertained of recovery.†

*Case* 3.—A clergyman, 48 years of age, of a stout athletic frame, and who had always enjoyed the most robust health, was affected with axillary aneurism, in consequence of a fall from his horse in December, 1812. In July following, the tumor was fully as large as a goose egg; on the sixteenth of that month Dr. Colles, of Dublin, secured the right subclavian artery with two ligatures on the acromial side of the scalene muscle. The time occupied by the operation is not stated. Death occurred on the fourth day from inflammation of the chest and limb.*

*Case* 4.—In 1815 Mr. Thomas Blizard secured the subclavian artery in the London Hospital. The case was one of aneurism in the left axilla; the ligature was applied at the usual point "with great facility;" and the patient died on the evening of the eighth day, previously to which event the ring and middle fingers turned black. On dissection the pericardium exhibited the effects of high inflammation; the heart, of a deep red colour on its posterior surface, was covered with flakes of lymph; the lining membrane of the aorta, right carotid and left subclavian was of a scarlet complexion, and the boundaries of the aneurismal tumor were in a state of sphacelation.†

*Case* 5.—This was the first case in which complete success attended the deligation of the subclavian artery, immediately after its exit from between the scalene muscles. The patient was a gentleman, with an aneurism in the left axilla. The tumor was of large size, and had encroached so much upon the clavicle as to throw it considerably above its natural level. The ligature was passed under the artery with great facility, and immediately on tying it all pulsation in the brachial and radial arteries ceased. In the afternoon the temperature of the limb was observed to be rather higher than that of the other arm. The operation was performed by the late Professor Post, of New York, on the 8th of September, 1817. On the 17th the aneurismal tumor burst, followed by a discharge of about three ounces of dark grumous blood. On the 26th the ligature was detached from the subclavian artery; and before the middle of October the wound was perfectly cicatrized.‡

*Case* 6.—On the 7th of March, 1819, Baron Dupuytren, surgeon to the Hotel-Dieu of Paris, tied the subclavian on the left side, just as it emerges from behind the scalene muscle, in a case of false aneurism of the axillary artery. The patient was of an excellent and robust constitution, thirty-seven years of age. Having been a soldier, he received, seven years ago, in a charge of cavalry, a thrust from a sword which penetrated the axillary artery. The tumor was about the size of a child's head

* Ramsden's Practical Observations on Surgery, p. 276

† Hodgson on the Diseases of the Arteries, &c.

* Edinb. Med. and Surg. Journal, vol. 2 p. 14.

† Hodgson, op. cit. p. 602.

‡ Cooper's Surgical Dictionary.

at the time of the operation, which lasted one hour and a quarter. About the fifteenth day the ligature came away; and on the 27th of March the wound was nearly healed. The patient made a perfect recovery.*

*Case* 7.—On the 30th of March, 1819, Dupuytren performed his second operation for axillary aneurism. The patient, formerly a soldier, forty years of age, and labouring under constitutional syphilis, received a wound from a flat-bladed lance in 1815. The tumor remained small for a considerable period, and then suddenly attained a great volume, extending itself under the pectoral muscle, and elevating the clavicle. The ligature was applied on the acromial margin of the scalene muscle, after one hour and forty-eight minutes; and the case terminated fatally on the ninth day, the swelling having been invaded by gangrene, and the patient having experienced several severe attacks of hæmorrhage from the wound.†

*Case* 8.—The next case is that of Robert Liston, Esq., of Edinburgh, who tied the subclavian artery on the third of April, 1820.—This was the first successful operation of the kind in Scotland. The patient, Alexander Gibson, a coachman, aged 35 years, referred the origin of his disease to a fall five months ago, in which he pitched on the left shoulder. Latterly the swelling increased with great rapidity, and seemed to extend above the clavicle, which it had very considerably displaced upwards. During the operation, which lasted altogether about half an hour, the anterior scalene muscle was divided. The ligature separated on the twelfth day, and the wound was completely cicatrized by the 8th of May, the tumor having in the meanwhile greatly diminished in size.‡

*Case* 9.—John Robertson, a porter, aged 47, was admitted into the Royal Infirmary of Edinburgh on the 7th August, 1823, for an aneurism of the left side, extending along the lower margin of the clavicle to the axilla. The tumor arose without any assignable cause, and when first noticed, about three weeks before, was scarcely larger than a bean. On the 23d, Wishart secured the subclavian artery external to the scalene muscle, the operation occupying only "a short time." On the sixteenth day after, the ligature was found lying loose in the wound; and a rapid recovery followed.§

*Case* 10.—In a patient upwards of 60 years of age, in St. Thomas' Hospital, Mr. Travers tied the subclavian artery, January, 17th, 1823, for axillary aneurism. The great elevation of the clavicle rendered the operation very difficult, independently of the profuse hæmorrhage. Altogether it occupied upwards of two hours. The patient died on the third day.*

*Case* 11.—George Vaughan, aged 36, a man of robust frame, an extra-tide waiter by occupation, lacerated his axillary artery in July, 1823, while making a sudden exertion with his arm. An aneurismal tumor rapidly formed, which by the end of August was about four inches in diameter, distinctly circumscribed, and extending from the lower edge of the clavicle to the lower border of the pectoral muscle. On the 20th of September, Charles Aston Key, Esq., of London, tied the right subclavian on the acromial side of the scalene muscle, the operation lasting twenty minutes. The ligature was detached on the twelfth day, and the patient speedily recovered. This appears to be the first case in which this vessel was secured with success in the London hospitals.†

*Case* 12.—Thomas Overall, brass-founder, aged 33, was admitted into St. Thomas' Hospital, August 1, 1825, under the care of Mr. Green, for aneurism of the right axillary artery. The tumor had existed upwards of five months, and was about the size of a large orange. His shoulder had been dislocated some time previously, and to that accident he attributed his present disease. "A long time elapsed in coming down to the artery," which was finally secured at the usual situation. Every thing went on well until the thirteenth day, when secondary hæmorrhage set in, which considerably weakened the patient, and rendered the success of the operation for a while doubtful. On the eighteenth day the wound was nearly healed, but the ligature had not yet separated from the artery.‡

*Case* 13.—John McIntyre, aged 43, presented himself to Mr. Liston, of Edinburgh, September, 1826, with a very large aneurismal tumor in the right axilla, equalling in volume a common-sized foot-ball. It passed upwards underneath the collar bone, which it had raised considerably, and downwards below the border of the axilla, pressing in the ribs and flattening the chest. About Christmas, 1825, he fell on the ice with his arm stretched out, and to this accident he ascribed his disease. On the 12th of September, Mr. Liston tied the subclavian artery at two points, the last close to the outer edge of the scalene muscle; and, although every thing promised well for a while, yet death occurred on the fourteenth day, preceded by slight hæmorrhage from the wound. On dissection the vessel was found to be considerably diseased; the wound was filled with coagula; and at neither of the points of deligation was there much appearance of reparation.§

* Edinburg Med. and Surg. Journal, vol. 16, p. 476.

† Edinburg Med. and Surg. Journal, vol. xvi. p. 199.

‡ Op. cit. vol. xvi, p. 348.

§ Op. cit. vol. xxi, p. 15.

* London Medico-Chir. Mag —Phila. Jour. Med. vol. vii, p. 183.

† Trans. of the Royal Med. and Chir. Society, vol xiii.

‡ Lancet, vol. viii, pp. 189-283.

§ Edinb. Med. and Surg. Jour., vol. xvii, p. 4.

*Case* 14.—Thomas Wharnby, aged 36, was admitted into the Manchester Infirmary, April 16, 1827, in consequence of an aneurismal tumor in the right axilla as large as a moderate sized orange. With the exception of this disease, which was first noticed about twelve months ago, he was apparently healthy. On the 17th of June, Robert Thorbe, Esq., tied the subclavian artery at the place usually selected for that purpose, the whole operation occupying fifteen minutes. The ligature separated on the 4th of July, and the patient got entirely well.*

*Case* 15.—William Wetson, wood-hawker, aged 38, a stout and very muscular man, was admitted into Guy's Hospital in November, 1827. The aneurism, which made its appearance without any assignable cause about nine weeks previously, occupied the right axilla, and was aboutthe size of an egg. The operation of tying the subclavian artery was performed, in the usual manner, by Bransby B. Cooper, Esq., on the 4th of December, and the patient had a speedy recovery.†

*Case* 16.—Professor Gibson of the University of Pennsylvania, tied the subclavian artery on the 17th of March, 1828, for axillary aneurism in John Langton, a muscular athletic man, a labourer by occupation, aged thirty-five. The disease was produced two days previously in an attempt to reduce an old luxation of the left shoulder-joint. The vessel was secured close to the outer margin of the scalene muscle; and the patient died on the 23d of March, the eighth day after the operation. The left hand and fore-arm were in a state of incipient gangrene, and no coagulum or trace of lymph could be observed above the ligature.‡

*Case* 17.—Hermenegildo Gonzales, a poor farmer, the father of three children, of small stature and spare habit, sixty-one years old, had always enjoyed good health until October 1827, when he began to feel pain along the course of the scalene muscles of the right side, which he supposed might have been occasioned by violent horse-back exercise. This was soon followed by the development of a tumour in the axilla, which by the early part of the following April was of a hemispherical form and measured three inches in diameter. The subclavian artery was tied by Dr. Edward W. Wells, of Maracaybo, on the 12th of April, on the acromial side of the scalene muscle; the ligature came away on the third of June, by which time the tumour had decreased to the size of a walnut; the wound was completely cicatrized by the fourteenth of that month; and the patient lived nearly three years after the operation, dying finally of ulceration of the bladder.§

* Medico-Chir. Review, January, 1828.

† London Med. and Phys. Jour., Feb., 1828.

‡ American Journal of the Medical Sciences, vol. ii, p. 136.

§ American Journal of the Medical Sciences, vol. iii. p. 28. See also vol. xiii, p. 406.

*Case* 18.—A stout muscular individual, a fisherman by occupation, aged forty-six, was admitted into the Parochial Infirmary at Davenport, England, June 10th, 1830, with a large, tense, pulsating tumour on the right side, extending from the cartilage of the fifth rib to the axilla on the point of the shoulder. It made its appearance about thirteen weeks previously, soon after an attack of rheumatism of the right arm; in a month it was of the size of a walnut, and in a fortnight thereafter it attained its greatest volume. On the 23d of June, Mr. Crossing tied the subclavian near its exit from between the scalene muscles, the whole operation lasting twenty-five minutes. The ligature came away on the *eighty-fifth* day, and in December following the tumour had nearly disappeared.*

*Case* 19.—William Hines, of Smithville, Virginia, labourer, twenty-eight years of age, became a patient of Professor Mott of New York, August 24th, 1830, for axillary aneurism of the right side. The tumour was about the volume of a goose-egg, and made its appearance seven weeks ago, soon after a violent strain produced by carrying a canoe on hand-bars across the arms. His general health was good. The artery was tied a little external to the scalene muscle, and at the expiration of twenty-seven days the wound was perfectly healed; the aneurismal tumour being very hard and much diminished in volume. The operation lasted fifteen minutes.†

*Case* 20.—The subject of this case was a man sixty-three years of age, who attributed his disease to a hurt received sometime before. The tumour, which resembled the section of an oblate spheroid, extended from the clavicle into the axilla of the right side, being about the size of a large orange. The operation of tying the subclavian artery was performed on the 17th of December, 1830, by W. Bland, Esq. of Australia; and the ligature fell off on the 29th of January, leaving the wound entirely healed.‡

*Case* 21.—In 1830, William H. Porter, Esq., of Dublin, tied the left subclavian successfully at the Meath Hospital, in a man forty years of age. The aneurismal tumor was situated at the part corresponding to the division between the great pectoral and deltoid muscles, had a firm pulsating feel, and was nearly ofthe volume of a goose-egg, having slightly displaced the clavicle. The ligature was applied at the usual point, and for about a month every thing progressed favourably. At the end of this time the tumour, which had diminished in size, began to increase and become painful, and, notwithstanding the diligent employment of anti-

* Medico Chirurg. Review, vol. xv. p. 507.

† American Journal of the Medical Sciences, vol. vii. p. 309.

‡ American Journal of the Medical Sciences, vol. ix. p, p. 527. See London Medical and Surgical Journal, Oct. 1831.

phlogistic measures, went on to suppuration. An incision being made into it, it discharged about a pint of fœtid purulent matter, mixed with large black clots of blood. Under the use of pressure it soon healed, and the man eventually recovered.*

*Case* 22.—In this case the artery was secured by Charles Mayo, Esq. of Winchester, England, March 26, 1831. The patient, W. Mathews, was forty-nine years of age, and the tumor, which was about six weeks' standing, occupied the left claviculo-axillary region. The ligation was completed in a little more than twenty minutes. On the 10th of May the wound was perfectly healed, and the tumour had disappeared.† Mr. Mayo operated, but unsuccessfully, on a similar case ten years before, the particulars of which I have not been able to obtain.

*Case* 23.—The next case of axillary aneurism in which the subclavian artery was tied, is that of William Ferguson, Esq. of Edinburgh. The patient, Mark Robson, a waiter, aged sixty, of intempeate habits, could assign no cause for the original appearance of the disease, but thought it might have resulted from some injury on the arm during one of his drunken fits. The tumour, which was scarcely perceptible for many months, rapidly increased within the last six weeks, and was now about the size of a large fist. The operation was performed in the usual manner on the 12th of May, 1831; the ligature separated on the 13th of June; and a rapid recovery followed.‡

*Case* 24.—Robert Blackwood, a weaver, aged sixty-five, was admitted into the Royal Infirmary of Glasgow in June, 1833, on account of a strongly pulsating tumour under the left clavicle, extending towards the lower part of the axilla. It was of an oblong, pyriform shape, measuring fully six inches in one direction by two and a half in the other. The swelling first appeared *above* the clavicle eighteen months ago, and, with the exception of cough and dyspnœa, both of which were aggravated on assuming the horizontal posture, the health of the patient was quite good. Dr. William Auchincloss tied the subclavian artery *within* the outer-third of the anterior scalene muscle, the operation, dressing and all, occupying about forty minutes. Death occurred in sixty-eight hours and a half, from effusion of serum on the surface and into the ventricles of the brain.§

*Case* 25.—Mrs. Hain, a weaver, of a spare habit of body, aged forty-two, had a pulsating tumor in the left axilla, which, on the 16th of April, 1834, was about the size of a man's fist. She could assign no cause for its origin, and first perceived it about ten years ago, when it was very small, and produced little or no inconvenience, even when engaged at her usual employment. Within the last six months it has increased with considerable rapidity, and eight days ago, when she experienced a sensation as if something had given way, she observed that the swelling had assumed a different shape and was much larger than previously. The local suffering also greatly augmented, and the general health became sensibly impaired. The subclavian artery was tied by Professor Lizars, of Edinburgh, on the 27th of April, on the acromial side of the anterior scalene muscle, in the short space of ten minutes. Scarcely any blood was lost; about sixty hours after the operation the pulsation could be felt in the arteries at the wrist; and the woman rapidly recovered without any untoward occurrence.*

*Case* 26.—The next case in which the subclavian was tied, and the last which I shall mention on this occasion, is that narrated by Dr. Samuel Hobart, of Cork. The tumor, which was situated immediately under the right clavicle, near its acromial extremity, was of the volume of a large duck-egg, and was first observed about four months previously, without any assignable cause. The patient, John Wright, aged 38, was "a respectable citizen of Cork, and of good bodily habits." The artery was readily secured on the acromial side of the scalene muscle on the 7th of May; the ligature was detached about the sixteenth day; and on the 30th of July the tumor had almost wholly disappeared.†

From the preceding abstract it will appear that there was only one female in the twenty-six cases. Axillary aneurism may therefore be regarded as being much more common in men than in women; agreeing, in this respect, with aneurism of the carotid, iliac, femoral, and popliteal arteries, as well as with that of the aorta.

Of these twenty-six patients, seventeen, or nearly two-thirds, were *cured*. Of the remaining nine, two died on the third day; one on the fifth; two on the fourth; two on the eighth; one on the ninth; and one on the fourteenth. The cause of death, as might be expected, was not alike in all the cases. In one—that of Dr. Auchincloss—it evidently resulted from the effusion of serum on the surface and into the cavities of the brain. In Professor Gibson's case, the left hand and fore-arm were in a state of incipient gangrene, and no effort had been made at reparation, although the patient lived until the eighth day. In John McIntyre, on whom Mr. Liston operated in 1826, slight hæmorrhage occurred a short time before death,

* Dublin Hospital Reports, vol. v. 1830. North American Medical and Surgical Journal, vol, xii. p. 106.

† Medico-Chir. Review, vol. xv. p. 482.

‡ Edinburgh Medical and Surgical Journal, vol. xxxvi. p. 309.

§ Edinburgh Medical and Surgical Journal, vol. xlv. p. 324.

* Lancet, 1834, vol. ii, p. 717.

† Edinb. Med. and Surg. Journal, vol. xlv. p. 48.

which happened on the fourteenth day; and on dissection it was found that the artery had given way in consequence of ulcerative absorption. In this case, too, scarcely any attempt had been made at restoration. In case vii, death occurred on the ninth day, preceded by several slight attacks of hemorrhage from the wound, and by gangrene of the aneurismal tumor. In the patient operated on by Dr. Colles, violent inflammation was discovered in the chest and limb; and in the case of Mr. Thomas Blizard, there were evidences of severe pericarditis and arteritis, with sphacelation of the aneurismal swelling and of the ring and middle fingers. In the other two cases no mention is made of the cause of death.

In ten of the cases, the disease occurred on the *left side*, in twelve on the right. The *youngest* of the patients was twenty-eight years of age; nine were between thirty and forty; eight between forty and fifty; and five between sixty and seventy; the oldest being sixty-five. In regard to their *occupation*, two were soldiers, one a gentleman, one a tailor, one a clergyman, one a coachman, one a farmer, one a wood-hawker, one a fisherman, one a tide-waiter, one a brass-founder, two weavers, two labourers, and two porters.

The *cause* of the disease is distinctly stated only in ten of the cases. In most it was produced by external violence, as a fall or strain. In the case of Professor Gibson, the tumor was developed soon after a fruitless attempt at reducing an old dislocation of the shoulder-joint; and in the soldiers operated on by Dupuytren the lesion was of a traumatic kind, the axillary artery being perforated by a sharp-pointed instrument. In one of the cases, that of a fisherman, forty-six years of age, the disease was attributed to the effects of rheumatism.

The *duration* of the disease varied from a few days to several years. In the case reported by Dr. Gibson, already alluded to, it was only of two days's standing; while in one of Dupuytren's it had existed for seven years, and for ten years in the one which fell under the observation of Professor Lizars. In this case, No. xiv, the aneurism came on without any assignable cause, and remained small until within six months of the time of the operation. In the other fourteen cases, in which I have been able to determine this point, the duration of the disease, prior to the deligation of the subclavian artery, was as follows:

In 1, 5 months; 1, 5 weeks; 1, 2 months; 1, 8½ months; 1, 12 months; 1, 10 weeks; 1, 7 months; 1, 7 weeks; 1, 3 months; 1, 6 weeks; 1, 18 months; 1, 4 months; 1, 4 years.

With respect to the *volume* of the aneurismal tumor, no definite information can be derived from the above cases, owing to the vague and unsatisfactory manner in which this subject has been treated by the reporters. Of the twenty-one cases in which this point is noticed, the following table may be presented, it being impossible to deduce any general conclusion from them.

In case 1, the tumor was "large;" in case 3, of the size of a goose-egg; in case 5, "large;" in case 6, child's head; in case 7, "very large;" in case 8, "large;" in case 10, "large;" in case 11, four inches in diameter; in case 12, very large; in case 13, foot-ball; in case 14, orange; in case 15, egg; in case 17, three inches in diameter; in case 18, "large;" in case 19, goose-egg; in case 20, large orange; in case 21, of the size of a goose-egg; in case 23, large fist; in case 24, six inches by two and a half; in case 25, fist; in case 26, duck-egg.

The length of time occupied in *performing the operation* is stated only in eleven cases, in which it varied from ten minutes, the minimum, to upwards of two hours, the maximum; the operator, in the former case, being Professor Lizars of Edinburgh, in the latter, Mr. Travers of London. Baron Dupuytren also consumed one hour and forty-eight minutes in one of his operations. In the remaining eight cases, the deligation of the subclavian occupied, in two, twenty minutes; in two, fifteen minutes; in one, twenty-five minutes; in one, forty minutes; and in two, thirty minutes.

In twelve of the cases the *ligature separated*, in three, on the twelfth day; in one, on the fifteenth; in two, on the sixteenth; in two, on the eighteenth; in one, on the thirty-second; in one, on the forty-third; and in one—that of Mr. Crossing—on the eighty-fifth.

